# Evolocumab-induced allergic reaction in a post-partial gastrectomy patient: A case report

**DOI:** 10.1097/MD.0000000000046793

**Published:** 2025-12-19

**Authors:** Xuxi Hong, Xuehui Jiang, Xiaohui Xu, Fangfang Xiong, Xiaowei Huang

**Affiliations:** a Department of Cardiology, Quanzhou First Hospital Affiliated to Fujian Medical University, Quanzhou, China; b Department of Pharmacy, Quanzhou First Hospital Affiliated to Fujian Medical University, Quanzhou, China; c Department of Orthopedics, Quanzhou First Hospital Affiliated to Fujian Medical University, Quanzhou, China; d Department of Pharmacy, The Second Affiliated Hospital of Fujian Medical University, Quanzhou, China.

**Keywords:** case report, drug allergy, evolocumab, PCSK9 inhibitor, post-gastrectomy

## Abstract

**Rationale:**

Evolocumab, a novel proprotein convertase subtilisin/kexin type 9 inhibitor, is widely used for intensive lipid-lowering therapy. However, its safety profile in patients with altered gastrointestinal anatomy (e.g., partial gastrectomy) remains inadequately characterized, particularly regarding allergic risks linked to immune microenvironment changes.

**Patient concerns:**

A 55-year-old male with coronary artery disease and a history of partial gastrectomy received subcutaneous evolocumab (140 mg) post-percutaneous coronary intervention. Within 24 hours, he developed a diffuse erythematous rash with pruritus on the chest and back.

**Diagnoses:**

Coronary artery disease (status post-percutaneous coronary intervention); allergic rash induced by evolocumab (Naranjo score: 7, WHO-UMC causality: probable).

**Interventions:**

Evolocumab was discontinued. Intravenous dexamethasone (5 mg/day) and oral cetirizine (10 mg/day) were administered for allergy control.

**Outcomes:**

Rash and pruritus significantly improved within 48 hours. One-week follow-up confirmed full resolution without hyperpigmentation.

**Lessons:**

Post-gastrectomy patients may face heightened evolocumab allergy risks due to immune dysregulation. Pre-administration assessment of surgical history and close post-dose monitoring are critical.

## 1. Introduction

Evolocumab is a novel medication designed to lower lipid levels. It is a fully human monoclonal antibody that focuses on proprotein convertase subtilisin/kexin type 9 (PCSK9). By interacting with the low-density lipoprotein (LDL) receptor, it leads to a substantial decrease in LDL-cholesterol (LDL-C) levels.^[1]^ Its effectiveness has been confirmed through numerous randomized controlled trials, indicating a reduction in LDL-C levels by 55% to 75% along with enhancements in various lipid profiles.^[2–5]^

Despite the overall favorable safety profile of this type of agent,^[6]^ it was suggested that it may induce allergic reactions.^[7]^ Notably, gastrointestinal surgeries (e.g., gastrectomy) may increase the risk of biologic-associated allergies by altering pharmacokinetics or the intestinal immune microenvironment.^[8]^

In patients after percutaneous coronary intervention (PCI), an intensive lipid-lowering regimen is often required in combination with PCSK9 inhibitors.^[9]^ However, there remains a paucity of safety evidence for the use of such agents in patients after gastrectomy, and the literature has not addressed the specific risks associated with the use of PCSK9 inhibitors in such patients.

## 2. Case report

### 2.1. Medical history and prior treatments

This case report was approved by the Institutional Ethics Committee of Quanzhou First Hospital Affiliated to Fujian Medical University (Approval No.: K062). Written informed consent was obtained from the patient for the publication and any accompanying images.

A male patient, aged 55, was hospitalized on June 12, 2025, due to a history of chest tightness and pain persisting for 10 years, which had reappeared 3 days earlier. His medical background shows coronary atherosclerotic heart disease, having undergone PCI in 2014 and 2015, along with hypertension for a decade, managed with perindopril and metoprolol. Additionally, he had a partial gastrectomy in 2017. He does not have any known allergies to medications and has been using aspirin, clopidogrel, and atorvastatin for a long time. When he was admitted, his vital signs were stable, displaying a temperature of 36.2°C, a pulse of 65 beats per minute, a respiratory rate of 20 breaths per minute, and a blood pressure of 115/70 mm Hg. Tests conducted on June 13, 2025, showed a weakly positive result for fecal occult blood, normal cardiac markers (TnI < 0.01 ng/mL), unremarkable D-dimer at 0.22 mg/L, potassium levels at 4.33 mmol/L, creatinine at 75 μmol/L, triglycerides at 1.85 mmol/L, LDL-C at 1.74 mmol/L, and high-density lipoprotein cholesterol at 0.46 mmol/L, with no additional significant abnormalities identified.

The patient was initially diagnosed with coronary atherosclerotic heart disease, hypertension, and postoperative status (partial gastrectomy). Then a preliminary treatment plan was formulated: medication therapy including Aspirin Enteric-Coated Tablets, Clopidogrel Tablets, Atorvastatin Calcium Tablets, Perindopril Tert-butylamine Tablets, Metoprolol Sustained-Release Tablets, and Pantoprazole Enteric-Coated Tablets were prescribed. Coronary angiography was planned, with PCI to be performed if necessary.

### 2.2. Surgery and postoperative treatment

On June 14, 2025, the patient had coronary angiography, which showed a 90% blockage in the stent of the left anterior descending artery, a 60% to 70% blockage in the obtuse marginal branch 1 of the left circumflex artery, and a 90% blockage in the stent of the right coronary artery. As a result, PCI was performed successfully, restoring blood flow to the targeted area.

On the 1st day after surgery, the antiplatelet treatment was modified by switching clopidogrel for ticagrelor 90 mg twice a day, while other medications stayed the same. By the 2nd postoperative day, a stronger lipid-lowering plan was started, which included an initial dose of evolocumab 140 mg given subcutaneously along with atorvastatin 20 mg to be taken every night.

### 2.3. Allergic reaction onset and management

**Figure 1.**
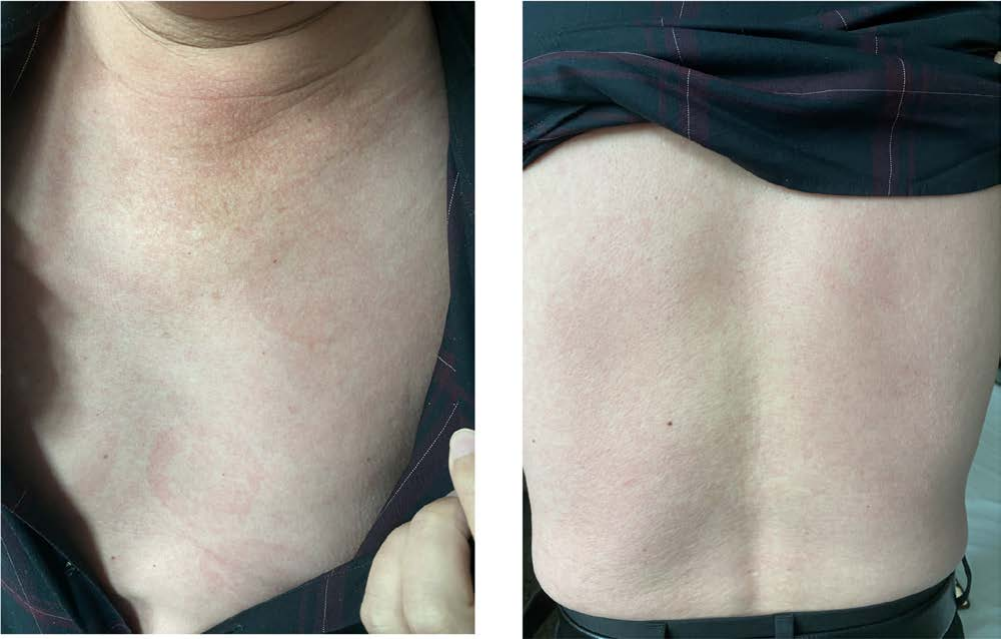
The allergic rash on the chest (left) and back (right) of the patient.

On postoperative day 3, within 24 hours after evolocumab administration, the patient developed a diffuse erythematous rash with pruritus involving the chest and back (Fig. 1), without respiratory symptoms or hemodynamic instability, and no recurrence of chest tightness or pain. Physical examination findings included a temperature of 36.5°C, blood pressure of 131/74 mm Hg, and an ill-defined urticarial rash without mucosal involvement. The laboratory tests revealed a mild elevation in eosinophils compared with prior levels. The evaluation of causality pointed to evolocumab as the primary suspected allergen. This conclusion was based on the clear temporal relationship (rash onset within 24 hours of administration) and the patient’s prior tolerance of ticagrelor, which had been administered 2 days earlier without any adverse effects. The treatment for the allergic reaction consisted of intravenous dexamethasone (5 mg/day) and oral cetirizine (10 mg/day). Ticagrelor was continued, as it was considered an unlikely cause of the rash.

### 2.4. Outcome and follow-up

The rash slowly decreased, and the itching became much less intense 48 hours after starting treatment for allergies. By the time the patient was ready to leave, the skin symptoms had significantly improved. The condition was eventually identified as an allergic reaction caused by evolocumab. In a follow-up visit 1 week after discharge, all the skin issues had healed completely without residual hyperpigmentation. The timeline of key events from drug administration to full recovery is provided in Table 1.

**Table 1:** Timeline of key events.

Date	Postoperative day	Event
June 14, 2025	Day 0	Coronary angiography and percutaneous coronary intervention performed.
June 15, 2025	Day 1	Antiplatelet therapy switched to ticagrelor.
June 16, 2025	Day 2	First subcutaneous dose of evolocumab (140 mg) administered.
June 17, 2025	Day 3	Diffuse erythematous rash with pruritus developed (within 24 h of evolocumab). Anti-allergy treatment (dexamethasone and cetirizine) initiated.
June 19, 2025	Day 5	Rash and pruritus significantly improved (within 48 h of treatment). The patient was discharged.
June 26, 2025	1-week post-discharge	Follow-up: complete resolution of skin lesions without hyperpigmentation.

## 3. Discussion

As new agents for lowering lipid levels, PCSK9 inhibitors effectively decrease LDL-C concentrations by blocking the breakdown of LDL receptors. This effect is especially notable in patients who either do not respond adequately to statins or cannot tolerate them.^[10]^ Evolocumab, a human monoclonal antibody, is thought to have low potential for immunogenicity. Nonetheless, this situation emphasizes the importance of monitoring for allergic reactions. Within a day of receiving the treatment, the patient experienced an allergic rash induced by evolocumab, with other medications and underlying conditions ruled out as causative factors. A limitation of this case is the absence of specific serum IgE testing to verify the allergic trigger.

This case has 3 key characteristics: the rash appeared within 24 hours after the initial dose; the patient’s previous partial gastrectomy might have changed the immune environment. It has been noted that surgery on the gastrointestinal system can affect the gut microbiota-immune connection and lead to a rise in T-helper 2 cytokines, increasing the possibility of allergic conditions;^[8]^ temporal analysis (reaction after new medications were given) and drug rechallenge testing (symptoms improved without stopping ticagrelor) ruled out other possible causes, thereby reinforcing the assessment of causation. This corresponds with the “Probable” classification according to the WHO-UMC criteria for assessing adverse drug reaction causality, with a Naranjo score of 7 (Probable).^[11]^ The adverse drug reaction probability scale was given in Table S1, Supplemental Digital Content, https://links.lww.com/MD/Q988.

Other potential causes for the rash were also carefully considered. Ticagrelor and iodinated contrast media were deemed unlikely, as the former was tolerated both before and after the reaction, and the latter typically causes immediate hypersensitivity or within a few hours of exposure. The strong temporal link exclusively to the 1st evolocumab dose therefore remains the most plausible explanation. Furthermore, the patient’s partial gastrectomy history is mechanistically relevant. Such gastrointestinal surgery can alter the gut microbiome–immune axis, potentially promoting a T-helper 2-polarized state that increases susceptibility to allergic reactions.^[8]^ This immune dysregulation may underlie the heightened hypersensitivity to an agent like evolocumab observed in this patient.

Despite the extremely low incidence of allergic reactions associated with evolocumab, real-world data suggest that vigilance to individual differences in clinical practice is still warranted. It is recommended that patients with hypersensitivity or the presence of abnormalities in immune regulation (e.g., autoimmune disease, transplantation history) be closely monitored after the first administration and that 1st aid equipment should be available.

This case has important implications for clinical practice. First, a detailed inquiry into the patient’s history of gastrointestinal surgery and allergies should be conducted before initiating biologic agents such as PCSK9 inhibitors, in order to identify individuals at potential high risk. Second, for patients with such a history, enhanced monitoring is recommended; the 1st dose should preferably be administered in a medical facility equipped with resuscitation capabilities. Third, in the event of rash occurrence, a stepwise management strategy is advised: for localized mild rashes, antihistamines with or without corticosteroids may be attempted without discontinuing the drug; for generalized urticaria or angioedema, evolocumab should be permanently discontinued and an emergency treatment protocol initiated. Finally, for patients requiring intensive lipid-lowering therapy who develop hypersensitivity to this agent, alternative medications with different mechanisms of action, such as inclisiran, may be considered. Its twice-yearly dosing schedule and unique small interfering RNA mechanism may help reduce the risk of allergic reactions.^[12]^ It is important to acknowledge the limitations of this case report. Firstly, as a single-case observation, its findings cannot be generalized to a broader population. Secondly, we did not perform long-term follow-up beyond the resolution of the acute rash. Despite these limitations, this case provides a valuable clinical alert.

In conclusion, this case confirms that evolocumab may induce an allergic reaction and a high level of vigilance is needed, especially in the presence of immunomodulatory abnormalities such as gastrointestinal surgeries. Medical institutions should establish a risk stratification system for the use of biological agents, and improve the pre-medication assessment and post-medication monitoring program for patients with allergic reactions.

## Author contributions

**Data curation:** Fangfang Xiong.

**Writing – original draft:** Xuxi Hong, Xuehui Jiang.

**Writing – review & editing:** Xiaohui Xu, Xiao-Wei Huang.

## Supplementary Material


