# Supplementary material for: Evolocumab-induced allergic reaction in a post-partial gastrectomy patient: A case report
Source: Medicine (Baltimore). 2025 Dec 19;104(51):e46793. doi: 10.1097/MD.0000000000046793 (PMC12727362; doi:10.1097/MD.0000000000046793)
Supplement: Supplementary file 1 [file medi-104-e46793-s001.docx]

| Questions | Yes | No | Do not know | Score |
| --- | --- | --- | --- | --- |
| 1. Are there previous conclusive reports on this reaction? | +1 | 0 | 0 | 1 |
| 2. Did the adverse event appear after the suspected drug was administered? | +2 | -1 | 0 | 2 |
| 3. Did the adverse reaction improve when the drug was discontinued or a specific antagonist was administered? | +1 | 0 | 0 | 1 |
| 4. Did the adverse reaction reappear when the drug was readministered? | +2 | -1 | 0 | 0 |
| 5. Did the adverse reaction reappear when the drug was readministered? | -1 | +2 | 0 | 2 |
| 6. Did the reaction reappear when a placebo was given? | -1 | +1 | 0 | 0 |
| 7. Was the drug detected in the blood (or other fluids) in concentrations known to be toxic? | +1 | 0 | 0 | 0 |
| 8. Was the reaction more severe when the dose was increased, or less severe when the dose was decreased? | +1 | 0 | 0 | 1 |
| 9. Was the reaction more severe when the dose was increased, or less severe when the dose was decreased? | +1 | 0 | 0 | 0 |
| 10. Was the adverse event confirmed by any objective evidence? | +1 | 0 | 0 | 0 |
| Total score |  |  |  | 7 |

**Supplementary Table 1** ADR probability scale
